# Acute and chronic dissection of pulmonary artery: new challenges in pulmonary arterial hypertension?

**DOI:** 10.1177/2045893217749114

**Published:** 2017-12-18

**Authors:** Michał Florczyk, Maria Wieteska, Marcin Kurzyna, Piotr Gościniak, Joanna Pepke-Żaba, Andrzej Biederman, Adam Torbicki

**Affiliations:** 1 Department of Pulmonary Circulation, Thromboembolic Diseases and Cardiology, European Health Centre Otwock, Centre of Postgraduate Medical Education, Warsaw, Poland; 2 Clinical and Invasive Cardiology Department, Maria Curie Skłodowska Province Hospital, Szczecin, Poland; 3 Pulmonary Vascular Diseases Unit, Papworth Hospital, Cambridge, UK; 4 Department of Cardiac Surgery, Medicover Hospital, Warsaw, Poland

**Keywords:** Pulmonary arterial hypertension, pulmonary artery dilatation, pulmonary artery dissection

## Abstract

Right ventricular failure is a leading cause of mortality in patients with pulmonary arterial hypertension (PAH). However, up to 25% of such patients die unexpectedly, without warning signs of hemodynamical decompensation. We previously documented that pulmonary artery (PA) dilatation significantly increases the risk of those deaths. Some of them may be due to dissection of PA resulting in cardiac tamponade. However, direct confirmation of this mechanism is difficult as most of such deaths occur outside hospitals. We present 4 patients with severe PAH and PA dilatation in whom PA dissection has been confirmed. Three patients had IPAH, one had PAH associated with congenital heart disease. All patients had mean pulmonary artery pressure (PAP) > 50 mmHg at diagnosis and dissection occurred late in the course of apparently well controlled disease (6 to 14 years). Several clinical elements were common to our patients - high systolic PAP, long lasting PH, progressive dilatation of PA to more than 50 mm with chest pain prior to dissection. However, clinical course followed three different patterns: sudden death due to cardiac tamponade, hemopericarditis caused by blood leaking from dissected aneurysm with imminent but not immediate cardiac tamponade, or chronic asymptomatic PA dissection. Indeed, two of our patients are alive and on lung transplantation waiting list for more than 2 years now. Further research is needed to suggest optimal management strategies for patients with stable PAH but significantly dilated proximal pulmonary arteries or confirmed PA dissection depending on the clinical presentation and expected outcome.

## Introduction

Right ventricular (RV) failure is a leading cause of mortality in patients with pulmonary arterial hypertension (PAH).^1,2^ However, up to 25% of such patients die unexpectedly, without warning signs of hemodynamic decompensation.^3,4^ We previously documented that pulmonary artery (PA) dilatation significantly increases the risk of those deaths.^4^ Some of them may be due to dissection of PA resulting in cardiac tamponade. Confirmation that pulmonary dissection may be one of the causes of unexpected death in PAH is difficult, as by definition, most of those deaths occur outside hospitals. On the other hand, dissection does not always lead to immediate death. Out of 63 cases of dissections of PA collected by Khattar et al.^5^ based on reports published up to 2003, eight have been diagnosed ante-mortem.^6–11^ We present four patients with severe PAH and PA dilatation in whom PA dissection has been confirmed. This represents 1.6% of 249 patients diagnosed with PAH during 1998–2009 and subsequently followed by our team.^4^ Based on this series, we try to identify clinical patterns which might indicate impending dissection. We also highlight individual differences in clinical presentation and outcome of PA dissection in PAH and discuss the available management strategies as well as challenges for the future.

## Case report 1

**Fig. 1.**
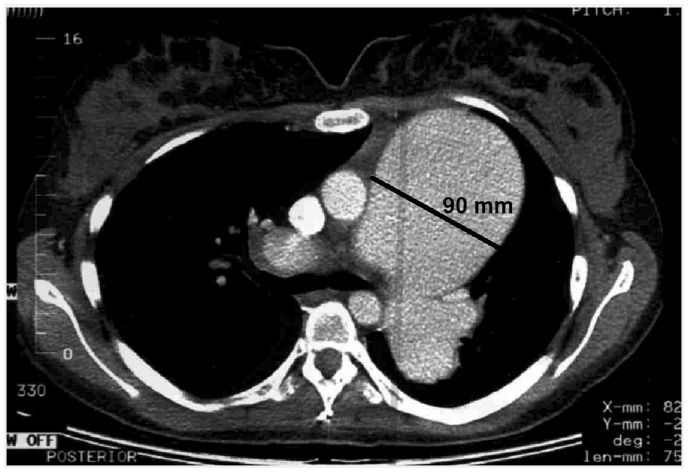
CT scan demonstrating an aneurysm of the pulmonary artery.

**Fig. 2.**
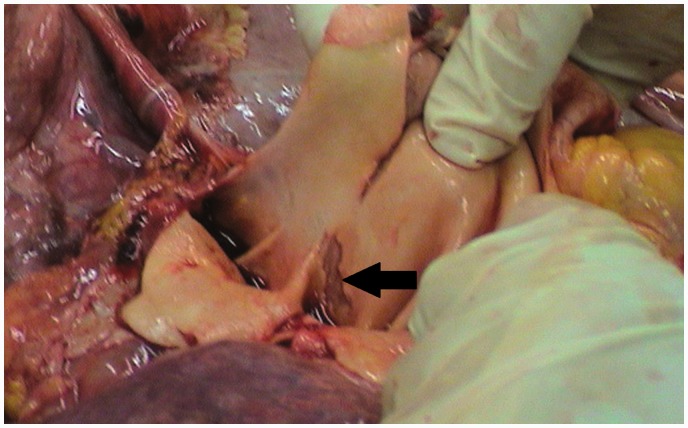
Postmortem examination revealed dissection (black arrow) of main pulmonary artery aneurysm.

A 22-year-old Caucasian woman underwent her first right heart catheterization (RHC) in May 2002. Six years earlier she had been suspected of having primary pulmonary hypertension (PH) based on non-invasive work-up in a pediatric center. One year later, pulmonary angiography revealed dilatation of the pulmonary trunk to 48 mm. Since that time, she had been on nifedipine 30 mg daily. At RHC we confirmed severely elevated pulmonary artery pressure (PAP) up to 90/40/62 mmHg, with high pulmonary vascular resistance (PVR) but preserved cardiac index (CI) (Table 1). On acute vasodilatory test, mean PAP (mPAP) fell below 40 mmHg indicating preserved vasoreactivity. By that time, the pulmonary trunk diameter increased to 80 mm as measured at magnetic resonance imaging (MRI). The dose of calcium channel blockers was increased to maximal tolerated dose (diltiazem 450 mg daily and amlodipine 5 mg daily) with echocardiographic and functional improvement. Because of the concerns related to PA aneurysm, the case was presented to an international group of experts. Lung transplantation (LTx) was considered premature in view of World Health Organization (WHO) functional class (FC) II and preserved vasoreactivity. The risk of hemodynamic decompensation after surgical reconstruction was considered excessive in view of expected abrupt reduction of “windkessel” proprieties of dilated proximal pulmonary arteries. This could further increase RV afterload in a patient already at high periprocedural risk due to severe PH. Maximal locally available targeted treatment was recommended and sildenafil 240 mg daily was added. Stable WHO FC II was maintained until six months later when the patient was admitted due to left-sided chest pain unrelated to respiration, which appeared one day before admission at rest and was accompanied by dyspnea. Further dilatation of the aneurysm up to 90 mm was found on computed tomography (CT) (Fig. 1). No signs of PA dissection could be found, but echocardiography revealed modest pericardial effusion, never observed earlier. The local surgical team found no possibility of intervention other than lung transplantation and the patient was put on the emergency list. Immobilization, sedation, and analgesics were introduced. A potential donor was identified on the fifth day but ultimately rejected. The patient died suddenly the next night, seven days after the onset of chest pain. Autopsy revealed PA dissection with cardiac tamponade as a cause of death (Fig. 2).

**Table 1.** Baseline characteristics and follow-up measurements of PAH patients who ultimately suffered from PA dissection.

	Case 1		Case 2		Case 3		Case 4	
Sex	F		F		F		F	
Etiology	IPAH (responder)		PAH-CHD		IPAH		IPAH	
Main symptom	Chest pain		Chest pain		Chest pain		Chest pain	
Outcome	Fatal		Alive		Alive		Fatal	
Follow-up from first assessment to dissection (years)	8		9		6		14	
Follow-up from dissection signs	7 days		5 years		4 years		Sudden death	
	At PAH diagnosis	Before dissection	At PAH diagnosis	Before dissection	At PAH diagnosis	Before dissection	At PAH diagnosis	Before dissection
Age (years)	15	23	38	47	40	46	37	51
PA diameter (mm)/ imaging method	80/MRI (48*)	90/CT	42/CT	54/CT	40/CT	57/CT	38/CT	66/CT
WHO FC	II	II	III	III	II	II	III	III
6MWT (m)	631	ND	412	ND	576	555	271	354
NT-pro-BNP (pg/mL)	69	ND	657	ND	1151	172	5816	122
PAP (s/d/m) (mmHg)	90/40/62	90/60/68	110/49/69	ND	102/57/78	112/42/67	81/44/56	93/44/59
mRAP (mmHg)	5	1	8	ND	3	4	11	1
CI (L/min*m^2^)	3.41	3.18	3.02	ND	2.68	2.57	1.6	1.81
PVR (WU)	8.5	11.1	12.7	ND	17.14	14.02	18.08	14.96

* Pulmonary aneurysm was revealed in pulmonary angiography 6 years before IPAH diagnosis.

IPAH, idiopathic pulmonary arterial hypertension; PAH, pulmonary arterial hypertension; CHD, congenital heart disease; PA, pulmonary artery; WHO FC, WHO functional class; 6MWT, 6-min walk test; NT-proBNP, N-terminal pro brain natriuretic peptide; PAP, pulmonary artery pressure; mRAP, mean right atrial pressure; CI, cardiac index; PVR, pulmonary vascular resistance; WU, Wood Unit; CT, computed tomography; MRI, magnetic resonance imaging.

## Case report 2

**Fig. 3.**
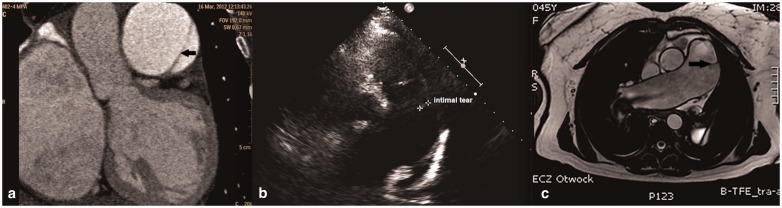
(a) CT, (b) echocardiography, and (c) MRI all revealed the intimal tear (black arrows) which began 1.5–3 cm beneath the pulmonary valve and was 3.5 cm long. The dissection extended slightly beyond the bifurcation of the main PA.

A 38-year-old Caucasian woman with Eisenmenger syndrome due to atrial septal defect (ASD) type II, was diagnosed to have PAH with mPAP of 69 mmHg (Table 1) and main PA aneurysm of 42 mm on CT performed in 2003. Several chronic mural thrombi were present in dilated central pulmonary arteries. Since 2004, the patient had been on endothelin receptor antagonist, remaining in WHO FC II. She was admitted in March 2012 after two episodes of pre-syncope. Several days earlier, she started to experience atypical chest pain. CT, echocardiography, and MRI (Fig. 3), all revealed dissection of the main PA which at that time was further dilated to 54 mm. CT angiography (CTA) also revealed compression of the left main coronary artery (LMCA) by the PA trunk and extrinsic proximal airway obstruction by dilated PA branches. Intrapulmonary thrombi were still present. While coronary angiography confirmed compression of the LMCA, it was not found hemodynamically significant on functional flow reserve assessment (FFR = 0.85). The patient was placed on a LTx list with intention of concomitant closure of ASD. Combination therapy with sildenafil was introduced, edema was controlled, and oral anticoagulation was changed to low molecular weight heparin. From 2012 to 2017, the patient remained on the LTx waiting list in stable WHO FC III without further significant PA dilatation or progressive dissection as assessed by repeated imaging.

## Case report 3

**Fig. 4.**
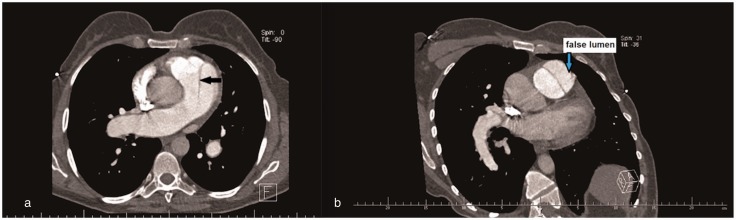
(a) CT scan showing dilated central pulmonary arteries with presence of a dissection flap in the main pulmonary artery (black arrow). (b) CT scan showing a linear dissection of the main pulmonary trunk with false lumen (blue arrow).

**Fig. 5.**
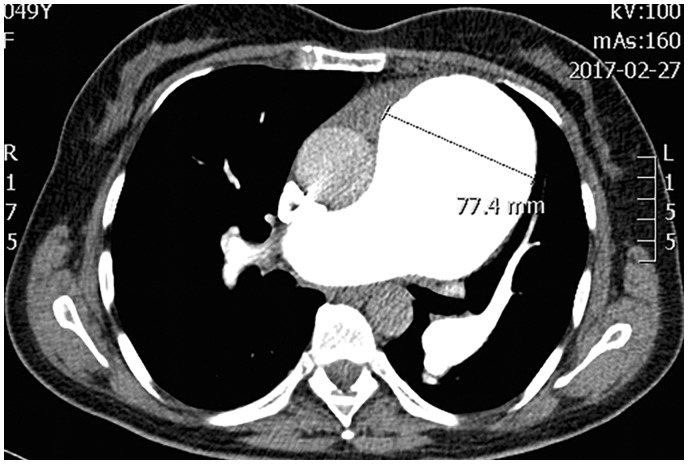
CT scan demonstrating further dilatation of the pulmonary artery to 77 mm.

A 40-year-old Caucasian woman was diagnosed with idiopathic PAH (IPAH) in 2007. RHC revealed high levels of PAP (mPAP = 78 mmHg), markedly increased PVR, but normal CI (Table 1). The patient was in WHO FC II. Dilatation of the main PA was already mentioned (40 mm). The patient received oral treprostinil, later combined with sildenafil. Patient remained in WHO FC II. At the beginning of 2013, CTA found no LMCA compression despite PA dilatation to 45 mm. In October 2013, the patient reported intermittent episodes of atypical chest pain not related to exercise. Transthoracic echocardiography revealed further dilatation of PA diameter to 57 mm. Emergency CTA revealed pulmonary artery dissection extending just above the pulmonary valve for 48 mm (Fig. 4). After consultations with cardiac surgical teams, the patient was listed for LTx. She remained on the active list from January 2014 to August 2017 without new symptoms, but further PA dilatation to 77 mm was found on CT (Fig. 5).

## Case report 4

A 37-year-old Caucasian woman with IPAH diagnosed in 2003, in WHO FC III, had dilatation of the main PA to 38 mm in CT. Sildenafil was started in 2003 and subcutaneous treprostinil was added in 2005 due to worsening of non-invasive prognostic markers. In October 2011, she started to complain of chest pain. CTA revealed compression of the LMCA, PA diameter increased to 49 mm without signs of dissection. A drug-eluting stent was implanted to the LMCA with resolution of chest pain. The patient remained in stable WHO FC III, but due to expanding PA aneurysm she was put on the active list for LTx. Over the next two years she remained stable, but CT revealed further PA dilatation: PA was 55 mm in February 2012, 55 mm in October 2012, 60 mm in June 2013, and 61.5 mm in September 2013. In January 2014, the patient moved to the UK and care was taken over by the National Pulmonary Hypertension Service Cambridge. In May 2014, CTA showed further PA dilatation to 66 mm, extending to both main branches. Over time, her treprostinil dose was increased to 54 ng/kg/min. In October 2016, her 6-min walk test (6MWT) improved to 354 m without desaturation while her exercise tolerance was limited by chest pain rather than dyspnea. Elective re-assessment of coronary arteries was considered. One month later she called an ambulance due to chest pain persisting for five days and she collapsed upon its arrival. She remained in cardiac arrest in spite of full life support with no obvious reversible cause. Autopsy revealed a massive 11-cm long fusiform aneurysm with a diameter of 10.5 cm involving the main pulmonary trunk and left main pulmonary artery. An intimal tear 7.5 cm long was noted at the junction of the main pulmonary trunk and left main pulmonary artery. There was a short dissection of the underlying wall as well as rupture into the pericardial cavity, causing massive hemopericardium with cardiac tamponade.

## Discussion

Prognostic implications of PA dilatation are not clear. Moderate increase of PA diameter above 29 mm, considered the upper limit of normal, is almost universally found in patients with PH and does not seem to affect outcome (12). More significant dilatations of central pulmonary artery have been associated with increased prevalence of unexpected deaths in hemodynamically stable patients with PAH or non-operable chronic thromboembolic pulmonary hypertension (CTEPH). In a group of 264 such patients followed for a median of 38 months, pulmonary artery diameter >48 mm measured with CT at diagnosis was related to a 7.5-fold increase in deaths which could not be unexplained by RV failure.^4^ Causes of those deaths, mostly occurring outside the hospital, are not easy to ascertain. They may be due to compression of the LMCA,^13^ potentially resulting in life-threatening ventricular arrhythmias induced by ischemia. Ventricular fibrillation was not a common finding among unselected patients with PAH who required resuscitation,^14^ but this may be different among patients at risk due to dilated pulmonary arteries. Intimal tear or rupture of dilated PA seems another likely life-threatening scenario.^15^ Increased availability of imaging methods offers more chance of monitoring PA dimensions as well as diagnosing PA dissection during lifetime.^16–20^ This, however, creates new problems regarding optimal management of individual patients. To better understand who could be in need of intervention, a comprehensive review of the literature has been undertaken recently to identify possible characteristics of high-risk PA aneurysms.^21^ Six trials collecting patients with PH assessed for the presence of PA aneurysm >40 mm were analyzed. PA aneurysms were found in 153 out of 1192 patients with PH. Concomitant analysis of case reports revealed 41 patients with PH in whom PA dissection was diagnosed. Thirty (73%) of those patients died, compared to 5/34 (15%) patients with pulmonary aneurysm but no dissection. Among patients with PH, dissections occurred most often—but not exclusively—in PAH associated with congenital heart disease (CHD). Mean PAP > 50 mmHg and longer duration of PH as well as PA diameter >75 mm and growth rate >2 mm/year identified patients as particularly high risk.^21^

Our case series of patients with PAH and PA dissection, the largest reported so far from a single group, seems to support these findings. Indeed, four presented cases represent 10% of all dissections reported so far in patients with PH. Our series also includes two patients who survived long term despite PA dissection, adding to only 11 patients with a similar outcome reported so far.^22,23^ Three patients had IPAH, one had PAH in the course of CHD. All patients had baseline mPAP > 50 mmHg and the interval between diagnosis and dissection was long (range = 6–14 years). At the time PAH had been suspected and/or diagnosed, the PA diameter did not exceed 48 mm in any of the patients and measured 48, 42, 40, and 38 mm, respectively. However, closer to or at the moment of diagnosis of dissection, all patients had a PA diameter of ≥48 mm (Table 1), indicating increased risk of sudden death, according to our previous report.^4^ On the other hand, only one out of four patients had a PA diameter >75 mm, which had been recently suggested by Duijnhover et al. as a clinically useful cut-off point for high-risk PA aneurysms.^21^ The mean annual growth of the PA diameter was in the range of 1.5–5.2 mm. Interestingly, three out of four patients had normal CI, despite high PVR, which resulted in very high systolic PAP since diagnosis (range = 81–110 mmHg). While one of the patients had significantly reduced CI and continued to be in WHO FC III, her right atrial pressure, NT-proBNP (N-terminal pro brain natriuretic peptide), and 6MWT were all better on follow-up than at the time of diagnosis, which would not be compatible with progressive RV failure. Many clinical elements were therefore common to all our patients: high systolic PAP; long-lasting PAH; progressive dilatation of PA; and chest pain before dissection. However, there were also significant differences in clinical course and outcome which followed three main patterns:

1. Sudden death due to cardiac tamponade within minutes to hours after dissection (exemplified by case 4). Preventive surgery of progressing PA dilatation seems the only effective management strategy. The type of surgical intervention—repair by graft, LTx, or heart–lung transplantation (HLTx)—should probably be selected according to severity of hemodynamic compromise, rate of progression of PA dilatation, and extension of PA aneurysm to distal arteries.^24^
2. Hemo-pericarditis caused by blood leaking from dissected aneurysm, with fatal cardiac tamponade within hours to days (case 1). Such a scenario might still offer a chance of emergency surgical intervention Replacement of the aneurysm with a graft even in hemodynamically compromised patients should be strongly considered. Stand-by ECMO support and concomitant listing for urgent LTx would be reasonable in such cases.^10^
3. Chronic PA dissection. This is the most surprising and rare scenario. In our two cases, dissection was preceded by atypical chest pain but not overt pericarditis. While we are witnessing relatively long survival in two of our patients, both are on the active waiting list for LTx. Although after dissection one would expect acceleration of diameter changes of PA, this is not always the case. Together with our patients, only 13 cases of chronic PA dissection have been reported in patients with PH.^21^ This is a new challenge for surgeons, who should consider graft implantation, only if the patient has a hemodynamic reserve high enough to survive the operation. Criteria which could assist in selecting between reconstructive surgery and waiting for LTx are not yet available.

## Limitations and controversies

Several smaller reports seem not to support the correlation of PA dilatation with sudden, unexpected death in PH. Badagliacca et al. followed 141 patients with PAH or CTEPH for a mean of 957 ± 438 days.^12^ Out of 47 deaths, 10% were sudden but occurred in patients with only mild to moderate PA dilatation (28–42 mm). Significant dilatation of PA seemed under-represented in this trial as only 2% and 0% of patients had a PA diameter above 45 mm and 55 mm, respectively. This is in contrast to our findings in which 29 (11%) and nine (3%) of 264 patients had PA diameters above those limits.^4^ Similar to us, Sakata et al. found a PA diameter of >50 mm in 12 (9%) and >60 mm in five (4%) of 130 patients with PAH. While mortality was significantly higher among 32 patients with PA diameter >40 mm compared with the remaining 98 patients (31% vs. 9%, *P* = 0.001), the authors attributed most deaths to RV failure, without supporting their opinion by autopsy data.^25^ Our observations directly confirm that PA dissection may represent a life-threatening complication of progressive PA aneurysm in patients with PAH. Interestingly, only one of our four patients had PAH associated to CHD leading to Eisenmenger syndrome, a common cause of PA dilatation. The current report does not include a fifth case of PA dissection with fatal cardiac tamponade, both confirmed at autopsy in a 66-year-old woman with a PA dilated to 61 mm at CT and echocardiographic signs of severe RV pressure overload. She died suddenly before RHC and differential diagnosis could be completed.

## Conclusions

PA dissection is an additional threat for prevalent, otherwise stable patients with longstanding PAH and preserved RV systolic function. Such patients may generate very high levels of pulmonary systolic pressure at rest and during exercise. Those with progressive PA dilatation should be actively followed. New chest pain may suggest imminent or ongoing PA dissection. In rare cases, PA dissection may not cause sudden death but may initially present as hemopericarditis, usually progressing to tamponade or—very rarely—result in chronic PA dissection. In all patients with PAH with imminent or confirmed dissection, urgent surgical intervention should be strongly considered. Further research is needed to suggest optimal management strategies for patients with PAH and significantly dilated proximal pulmonary arteries. Extrapolating criteria derived from aortic aneurysms, as recently suggested, seems an over-simplification.^26^
